# Prevalence of HIV, HBV and chlamydia infections in Cameroonian University context: case of the University of Dschang, in the Western region

**DOI:** 10.1186/1471-2334-14-S2-P3

**Published:** 2014-05-23

**Authors:** Martin Sanou Sobze, Jean Hubert Donfack, James-Francis Onohiol, Joseph Fokam, Ghislaine Bruna Djeunang Dongho, Patrick Stephane Nkamedjie Pete, Vittorio Colizzi, Gianluca Russo

**Affiliations:** 1 Department of Biomedical Sciences, Faculty of Sciences, University of Dschang, Dschang, Cameroon

## Introduction

In sub-Saharan Africa HIV infection remains largely epidemic, whereas HBV infection is highly endemic (>8%). In Cameroon, the prevalence of HIV is 4.3%. Concerning HBV and chlamydia infections, their prevalence are both ≥10%. Teenagers and young adults, including university students, are the population groups mostly affected by STDs. Epidemiological data on these infections, among university students could be helpful to implement specific prevention strategies.

## Methods

A descriptive study was performed in May 2013 among 624 students from the University of Dschang, Cameroon. Participants were screened for HIV, HBV and Chlamydia infections. Data was collected by a standard questionnaire and analyzed by Epi Info.

## Results

The average age of participants was 23.3 years (σ = 3.2) with a female predominance (58.7%). The observed prevalence of HIV, HBV and Chlamydia infection was 1.1% (7/624), 2.8% (5/176) and 2.0% (2/100) respectively. 83.2% of participants were sexually active. Concerning sexual risky behaviors, participants reported having multi partners (14.8%), using condom regularly (36.4%), occasionally (58.6%) or never (5.0%). 100%, 62.6% and 52.2% reported to be aware on HIV, HBV and Chlamydia infections respectively. In addition, only 5.5% and 21.3% of the participants were aware of their HBV and Chlamydia status respectively, versus 64.4% for HIV. The excessive cost of HBV and Chlamydia tests has been identified as the major barrier to testing (87.6%).

## Conclusion

Among college Cameroonian students the prevalence of HIV, HBV and Chlamydia infections seems to be relatively low if compared to general population. However, having multiple sexual partners in addition to non-systematic use of condoms during sexual intercourse represents risky behaviors among students. Awareness campaigns and screening facilitation on HBV and chlamydia infections among students need to be strengthened.

